# Unexpected metachronous multiple primary cancers: pilonidal sinus‑related squamous cell carcinoma and incidentally detected gastric gastrointestinal stromal tumour

**DOI:** 10.1093/bjrcr/uaaf065

**Published:** 2025-12-09

**Authors:** Carla-Ioana Hurjui, Sorinel Lunca, Anca Munteanu

**Affiliations:** Radiation Oncology Department, Regional Institute of Oncology, Iasi, Iasi County, 700483, Romania; Second Surgical Department, Regional Institute of Oncology, Iasi, Iasi County, 700483, Romania; Grigore T. Popa University of Medicine and Pharmacy Surgery Department, Iasi, Iasi County, 700115, Romania; Radiation Oncology Department, Regional Institute of Oncology, Iasi, Iasi County, 700483, Romania; Grigore T. Popa University of Medicine and Pharmacy, Third Medical Sciences Department, Iasi, Iasi County, 700115, Romania

**Keywords:** pilonidal abscesses, squamocellular carcinoma, radiation oncology, gastrointestinal stromal tumor

## Abstract

This case report presents a 62‑year‑old male with chronic pilonidal disease that underwent malignant transformation into squamous cell carcinoma (SCC), followed 18 months later by incidental detection of a gastric gastrointestinal stromal tumour (GIST) during routine oncologic surveillance. Initial contrast‑enhanced CT revealed the gluteal SCC, which was surgically excised and treated with adjuvant radiotherapy. Subsequent CT scan indicates a well‑circumscribed mass in the submucosa of the stomach. MRI and endoscopic biopsy confirmed a submucosal gastric GIST, which was completely resected. No recurrence of either malignancy has been observed on follow‑up. This case underscores the pivotal role of multimodality imaging in diagnosis, staging, and longitudinal care.

## Background

Pilonidal disease most commonly affects young adult males and typically presents as a cyst or abscess near the sacrococcygeal area.^1^ While generally benign, chronic or neglected pilonidal disease can very rarely progress to squamous cell carcinoma.^2^ Gastrointestinal stromal tumours (GISTs) are uncommon mesenchymal neoplasms of the gastrointestinal tract, usually discovered incidentally on imaging.^3^ We report an unusual case of dual malignancy, highlighting the diagnostic value of radiology and the need for vigilant follow‑up.

## Case report

A 62‑year‑old Caucasian male with type 2 diabetes mellitus, hypertension, dyslipidemia, and a history of recurrent pilonidal abscesses (treated surgically in 1980 and 1985) presented with a firm subcutaneous mass in the right buttock.

**Figure 1.**
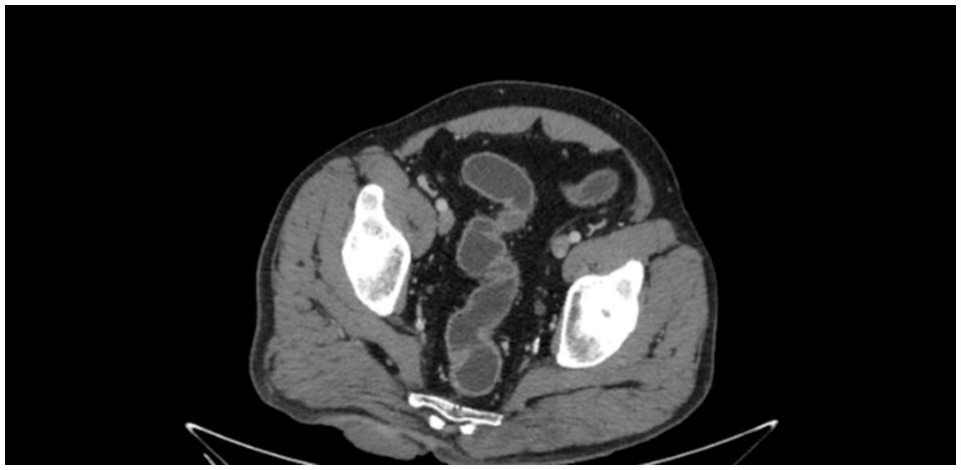
The current patient has underwent an axial CT showing subcutaneous gluteal mass with overlying skin thickening and no muscular invasion. This image was captured during a CT scan the patient has underwent in the Institute.

Contrast‑enhanced CT (Figure 1) demonstrated a 5.2 × 2.0 cm enhancing subcutaneous lesion with overlying skin infiltration but no muscular or osseous invasion. Wide local excision confirmed a well‑differentiated verrucous SCC (pT3Nx, G1, L0V0Pn0)- performed on 15th February 2022.

**Figure 2.**
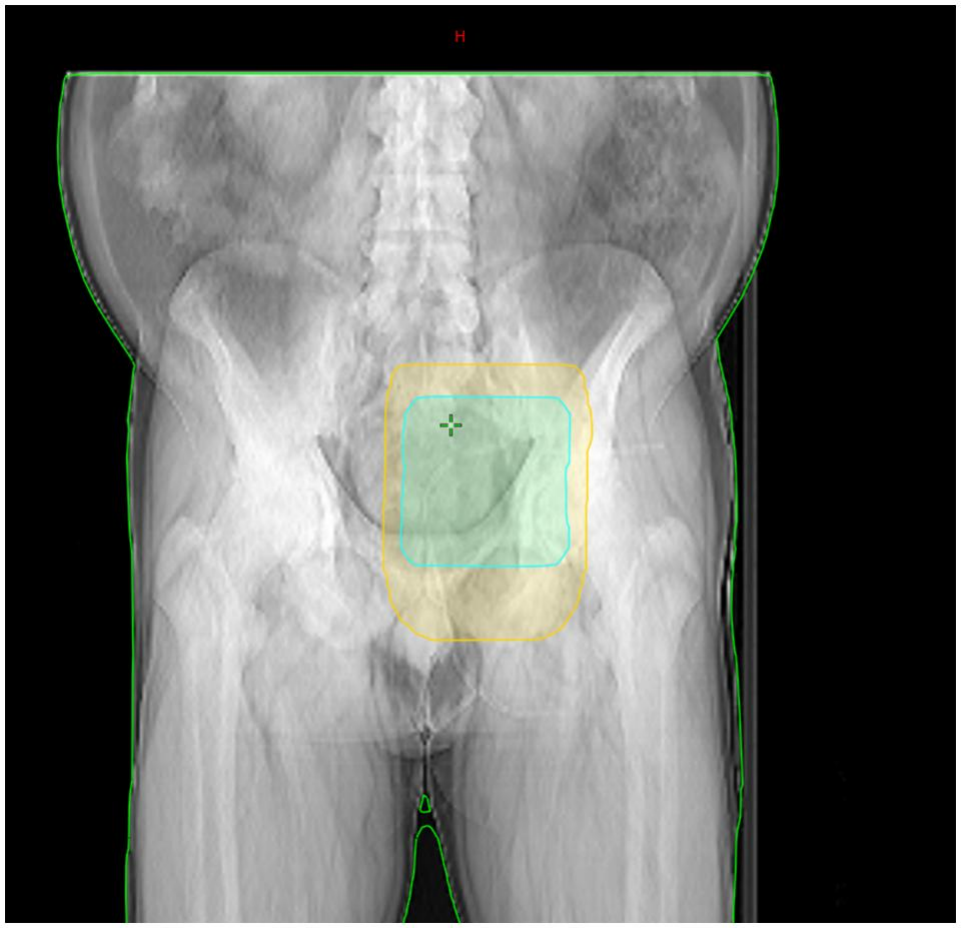
Coronal computer tomography view of the irradiated tumoral bed; the PTV (planning target volume) is marked in yellow, while the PTV boost can be observed in teal (patient in prone position).

Between 2nd August and 22nd September 2022, the patient is directed to the Radiotherapy Department of the Institute, where he has been administered adjuvant external-beam radiotherapy with photon beam of 6 MV, through intensity-modulated radiotherapy (IMRT) technique to the Clinac iX Linear Accelerator, in a total dose of 50 Gy in 25 fractions, with 2 Gy per fraction to the PTV right buttock and simultaneous integrated boost (SIB) up to 60 Gy in 25 fractions with 2.4 Gy per fraction to the tumour bed. To optimize the dose distribution in the target volume, a 0.5 cm bolus was placed (Figure 2).

**Figure 3.**
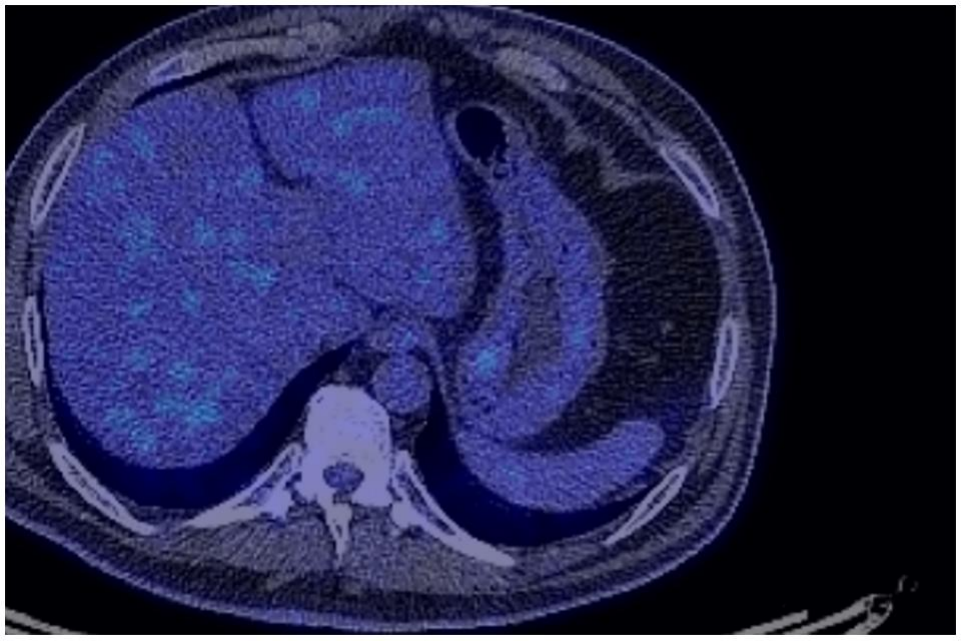
Axial fused PET‑CT demonstrating no abnormal focal FDG uptake.

**Figure 4.**
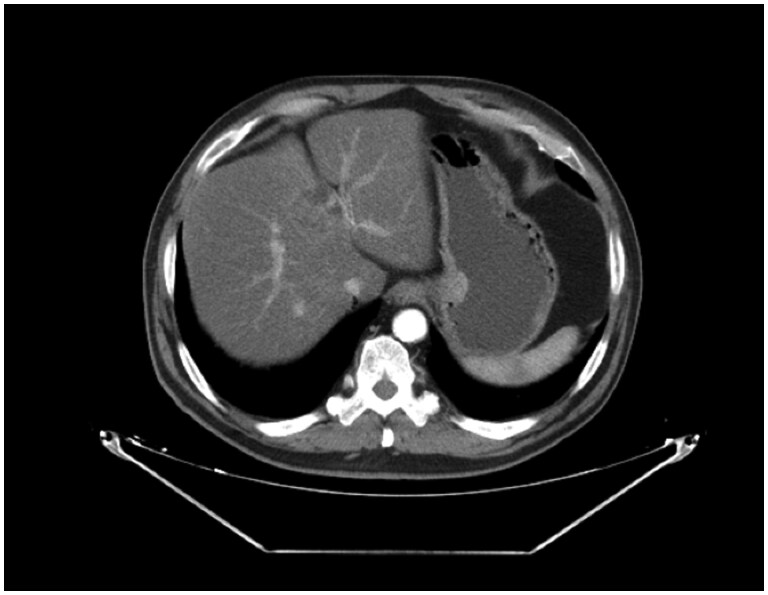
Axial contrast-enhanced CT-scan slice showing a well‑circumscribed submucosal mass on the lesser curvature of the stomach.

**Figure 5.**
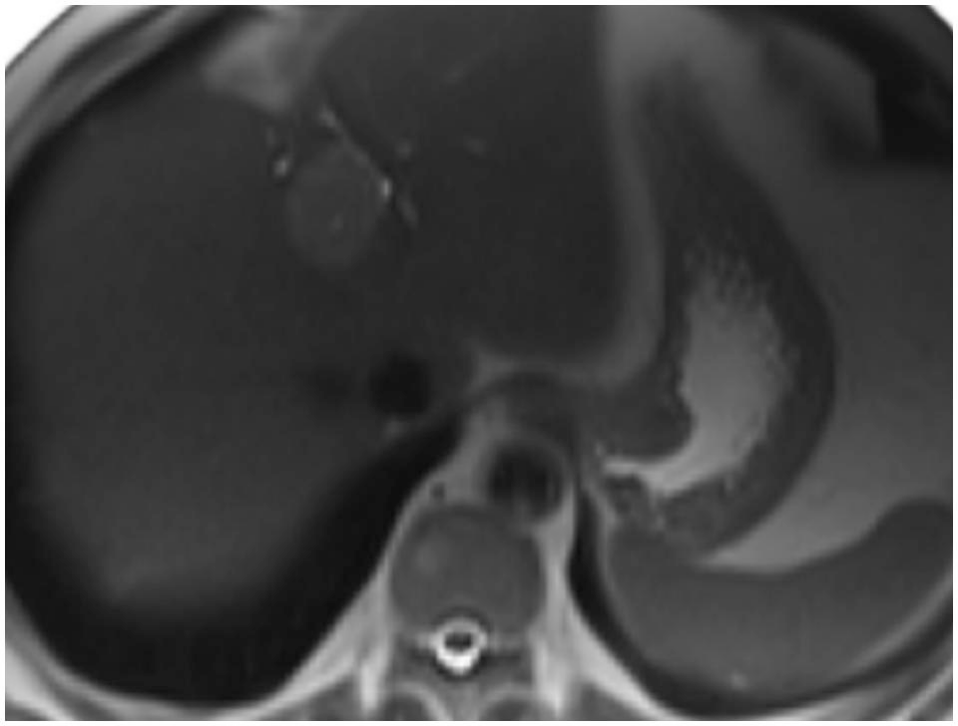
Axial MRI slice showing a well‑circumscribed submucosal mass on the lesser curvature.

**Figure 6.**
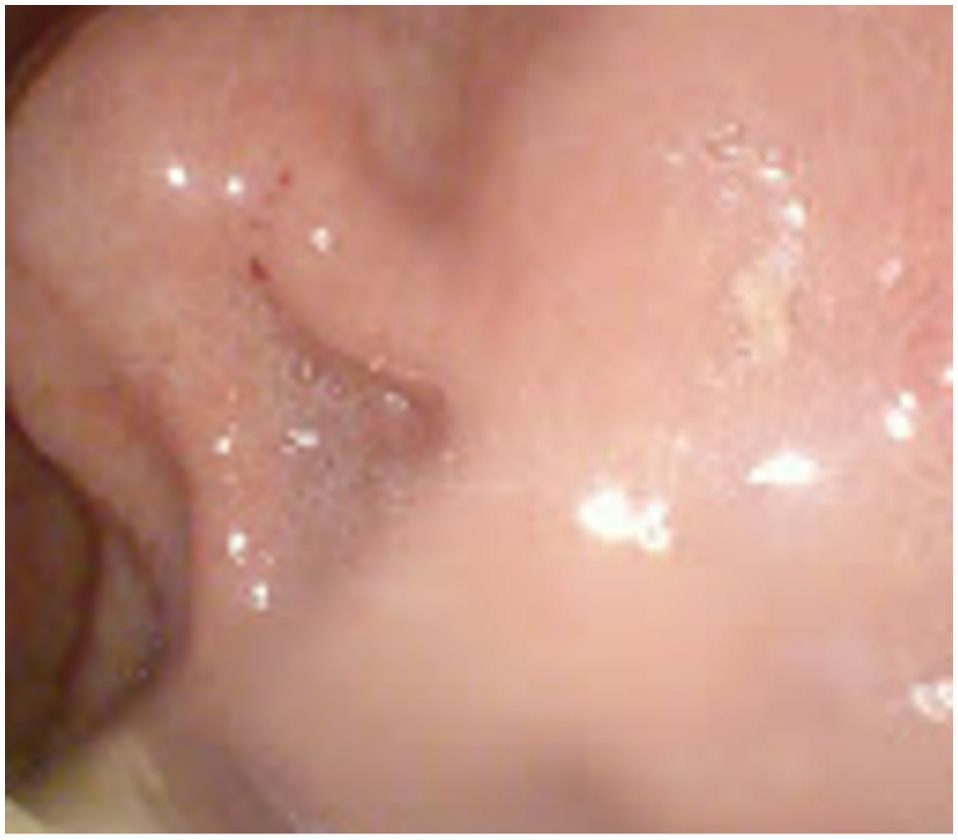
Endoscopic view confirming a submucosal lesion bulging into the gastric lumen.

**Figure 7.**
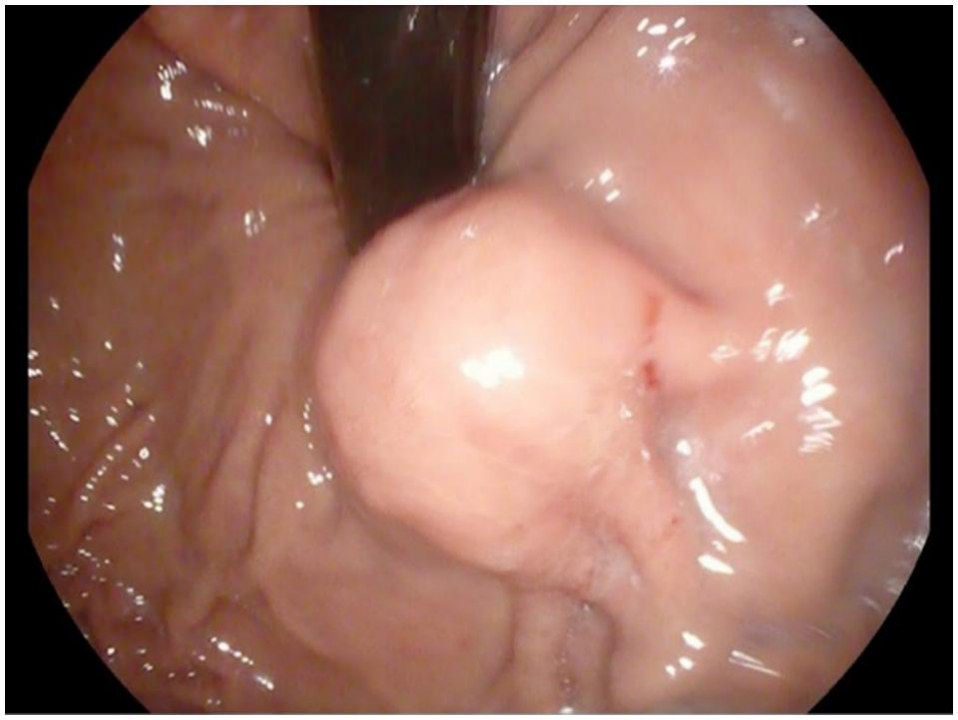
Distinct angle of the same upper-gastrointestinal tract endoscopy, confirming a submucosal lesion bulging into the gastric lumen.

A surveillance PET‑CT performed on 16th May 2022 showed no abnormal focal FDG uptake. (Figure 3). The following surveillance CT scan performed on 4th September 2023, however, indicates a well‑circumscribed gastric submucosal mass that requires further medical investigations. (Figure 4). Axial MRI further delineated a well‑circumscribed submucosal mass measuring 18 mm on the lesser curvature (Figure 5). Upper endoscopy revealed a submucosal bulge, and biopsy confirmed a gastric GIST (Figures 6 and 7).

The patient underwent laparoscopic wedge resection; histopathological examination confirmed a low‑risk gastric GIST (pT1Nx, G1, L0V0Pn0). Six‑month follow‑up MRI and endoscopy showed no residual or recurrent disease.

## Discussion

Malignant transformation of pilonidal disease is exceedingly rare, with fewer than 100 cases reported. Typical risk factors include long‑standing, recurrent infection and inadequate surgical management.^1^^,^^2^ In our patient, early cross‑sectional imaging facilitated prompt surgical treatment and adjuvant radiotherapy, achieving durable local control.

GISTs arise from the interstitial cells of Cajal and constitute the most common mesenchymal tumours of the gastrointestinal tract. They frequently present as incidental findings on imaging obtained for unrelated conditions.^3^ In this case, incidental PET‑CT unexpected gastric uptake triggered targeted endoscopy and MRI, allowing curative resection at an early stage.

No causal link between pilonidal‑associated SCC and gastric GIST has been established. Their coexistence likely reflects coincidental malignancies compounded by the patient’s diabetic pro‑inflammatory state.^4^^,^^5^ Nevertheless, the case illustrates the importance of comprehensive imaging follow‑up in complex oncology patients.

## Conclusion

Multimodality imaging enabled timely diagnosis, staging, and successful management of two rare and unrelated malignancies in the same patient. Clinicians should maintain a high index of suspicion for malignant transformation in chronic pilonidal disease and remain attentive to incidental findings that may signify additional neoplasms.

## Learning points

- Metachronous multiple primary cancers are described as the occurrence of multiple malignancies that develop from different tissues with distinct morphologies after a period of more than 6 months between them.
- Chronic, recurrent pilonidal disease can rarely (<0.1%) transform into squamous cell carcinoma.
- Multimodality imaging (CT, PET‑CT, MRI) is essential for detecting, characterising, and monitoring rare tumours.
- Gastrointestinal stromal tumours are frequently incidental radiologic findings requiring histological confirmation.
- There is no established pathological link between SCC and GIST; their coexistence in this patient is likely coincidental.
- Type 2 diabetes mellitus may increase overall cancer risk via chronic inflammation and impaired tissue repair.
